# Enhanced brain tumor diagnosis using combined deep learning models and weight selection technique

**DOI:** 10.3389/fninf.2024.1444650

**Published:** 2024-11-26

**Authors:** Karim Gasmi, Najib Ben Aoun, Khalaf Alsalem, Ibtihel Ben Ltaifa, Ibrahim Alrashdi, Lassaad Ben Ammar, Manel Mrabet, Abdulaziz Shehab

**Affiliations:** ^1^Department of Computer Science, College of Computer and Information Sciences, Jouf University, Sakkaka, Saudi Arabia; ^2^College of Computing and Information, Al-Baha University, Alaqiq, Saudi Arabia; ^3^REGIM-Lab: Research Groups in Intelligent Machines, National School of Engineers of Sfax (ENIS), University of Sfax, Sfax, Tunisia; ^4^Department of Information Systems, College of Computer and Information Sciences, Jouf University, Sakaka, Saudi Arabia; ^5^STIH: Sens Texte Informatique Histoire, Sorbonne University, Paris, France; ^6^Prince Sattam bin Abdulaziz University, Al-Kharj, Saudi Arabia

**Keywords:** brain tumor prediction, ensemble learning, vision transformer, genetic algorithm, parameter selection

## Abstract

Brain tumor classification is a critical task in medical imaging, as accurate diagnosis directly influences treatment planning and patient outcomes. Traditional methods often fall short in achieving the required precision due to the complex and heterogeneous nature of brain tumors. In this study, we propose an innovative approach to brain tumor multi-classification by leveraging an ensemble learning method that combines advanced deep learning models with an optimal weighting strategy. Our methodology integrates Vision Transformers (ViT) and EfficientNet-V2 models, both renowned for their powerful feature extraction capabilities in medical imaging. This model enhances the feature extraction step by capturing both global and local features, thanks to the combination of different deep learning models with the ViT model. These models are then combined using a weighted ensemble approach, where each model's prediction is assigned a weight. To optimize these weights, we employ a genetic algorithm, which iteratively selects the best weight combinations to maximize classification accuracy. We trained and validated our ensemble model using a well-curated dataset comprising labeled brain MRI images. The model's performance was benchmarked against standalone ViT and EfficientNet-V2 models, as well as other traditional classifiers. The ensemble approach achieved a notable improvement in classification accuracy, precision, recall, and F1-score compared to individual models. Specifically, our model attained an accuracy rate of 95%, significantly outperforming existing methods. This study underscores the potential of combining advanced deep learning models with a genetic algorithm-optimized weighting strategy to tackle complex medical classification tasks. The enhanced diagnostic precision offered by our ensemble model can lead to better-informed clinical decisions, ultimately improving patient outcomes. Furthermore, our approach can be generalized to other medical imaging classification problems, paving the way for broader applications of AI in healthcare. This advancement in brain tumor classification contributes valuable insights to the field of medical AI, supporting the ongoing efforts to integrate advanced computational tools in clinical practice.

## 1 Introduction

Brain tumor is the most prevalent condition in children and also the most challenging sickness to identify. Despite the advancements in technology and the vast research being conducted to detect and categorize brain tumors, it remains a difficult endeavor due to the varied appearance of tumors and their similarity to normal brain structures. Magnetic resonance imaging (MRI) is the recommended modality for the detection of brain tumors. This procedure is remarkably effective despite being quite time-consuming, and the results are of great quality. The main goal of this study is to identify and categorize the tumor based on the provided MRI imaging. The implementation of automated tumor detection algorithms on MRI images would facilitate the identification of tumors at the earliest possible stage, a critical factor in the treatment of brain tumors. Once the tumor is detected, it is classified according to its severity. This task is intricate and requires a significant amount of time, as it is performed by radiologists who analyze a vast collection of MRI images. The implementation of automated brain tumor identification and classification will mitigate human error and expedite the detection process. The initial step in detecting and classifying brain tumors is to establish a system that assists in segmenting MRI images. This stage include the initial processing of MRI images, the extraction and reduction of features, and the classification of the tumor. The next step of the research is assisting the radiologist with the extensive database. Automated brain tumor classification is essential for alleviating the burden on radiologists and offering an effective tool for tumor categorization.

Brain tumor classification is frequently discussed to assist radiologists in accurately interpreting brain MRI images for diagnosis (Zahid et al., 2022). Prior research has demonstrated the enhancement of different cutting-edge deep learning models in the categorization of brain tumors. Post-secondary tests and categorization rely on achieving accuracy and evaluating image quality, which is a laborious task. Upon the object's discovery, it is imperative to promptly identify all potential attributes and connections among its fundamental elements. This is a broad and abstract idea. Convolutional neural networks have demonstrated success in image processing applications within the realm of deep learning models. The typical architecture comprises many convolutional layers. These layers capture and isolate different characteristics found in the input image. However, these qualities can be more accurately detected by further discerning the most crucial characteristics of the layers. The pooling layer is typically used to achieve significant features, but in the current day, there is a pressing need to identify important features at each layer.

Classical machine learning methods have demonstrated little efficacy in addressing real-world problems across several domains, despite their ability to generate scores for tasks using predefined knowledge sets. Furthermore, the process of extracting features from the highly-dimensional inputs of advanced medical devices becomes insignificant when compared to the constant emergence of new techniques in the field of deep learning. Hence, in this study, ensemble learning methods are favored since they allow for the simultaneous utilization of two or more classifiers and generally exhibit superior performance compared to deep learning algorithms. This work introduces a new ensemble learning technique dubbed BT-ViTEff, specifically developed for the classification of brain tumors in medical MRI scans. The approach seeks to address difficult limits, such as intricate and ever-changing backgrounds, which arise due to the presence of varying backgrounds in the input medical photos. ViTEff combines the Convolutional Neural Network EfficientNet v2 (EfficientNetV2) with the vision transformer V2 model. EfficientNet v2 is a newly developed convolutional neural network (CNN) structure. The approach seeks to address the training limitation of EfficientNet models by showcasing enhanced parameter efficiency and accelerated learning speed as compared to similar models. The first steps of preprocessing consist of resizing the receiving MRI images to dimensions of 224 × 224 pixels. Afterwards, a range of data augmentation techniques, including rotation, shearing, shifting, and zooming, are used to enhance the diversity of the training data. This improves the capacity of our model to precisely categorize brain tumors into 44 different groups and avoids overfitting. Afterwards, the MRI images are fed into the EfficientNet v2 and ViT v2 models to extract complex characteristics, resulting in a comprehensive representation of various brain tumor classifications. The fusion model utilizes Weighted Average Ensembling and Simple Average Ensembling approaches to combine the feature maps produced by the EfficientNet v2 and ViT v2 models. Ultimately, we utilize the genetic algorithm to establish the optimal and superior weight for each deep learning model employed.

The subsequent sections of the paper are structured in the following manner. Section 2 provides an overview of previous studies, while Section 3 provides a detailed explanation of the materials and procedures used in our research. The experimental findings are analyzed in Section 4, and Section 5 presents the final conclusions of this study.

## 2 Related work

Recent advances in medical image classification have leveraged various machine learning and deep learning models to enhance diagnostic accuracy. Studies have shown that ensemble learning, which combines multiple model predictions, often outperforms individual models by capturing complementary strengths. In this section, we aim to describe same previous work proposed.

### 2.1 Traditional machine learning techniques

Various machine learning models, including decision tree, K-nearest neighbor (K-NN), logistic regression, and multiple support vector machine (SVM) models, were created using the suggested features to classify brain tumors.

Wisaeng and Sa-Ngiamvibool (2023) proposed a novel method, known as fuzzy Otsu thresholding morphological approach, for segmenting brain tumors. The values derived from each histogram in the original MRI image were modified by the implementation of a color normalizing preprocessing approach, together with histogram specification. The data indicates that the accuracy rates for images of gliomas, meningiomas, and pituitaries are 93.77 percent, 94.32 percent, and 94.37 percent, respectively. This unequivocally confirms that these occurrences can be precisely identified.

Jena et al. (2022) introduced a method for classifying and segmenting brain tumors by utilizing textural data and employing various machine learning methods. The technique is comprised of two distinct stages: tumor categorization and tumor segmentation. During the tumor classification stage, the MRI scans undergo pre-processing, and texture features are taken from the images using several techniques for texture extraction. The retrieved features were merged to create a feature vector matrix with dimensions of 200 × 471. Afterwards, the feature vector matrix was employed to train five machine learning algorithms: Support Vector Machines (SVM), k-Nearest Neighbors (k-NN), binary decision trees, Random Forest (RF), and ensemble approaches. The experimental findings indicate that the ensemble approaches yielded the most favorable outcome, attaining a classification accuracy of 96.98% and 97.01% for BraTS2017 + TCIA and BraTS2019 + TCIA, respectively.

Varuna Shree and Kumar (2018) have proposed a method that entails the classification of a tumor into one of two categories: “malignant” or “benign.” To extract features, they employ the discrete wavelet transform, and they apply a Support Vector Machine (SVM) for classification. Compared to the other classification algorithms, the Support Vector Machine (SVM) consistently outperformed them in terms of accuracy.

Furthermore, Dev et al. (2019) developed a novel approach to differentiate between malignant and non-cancerous tumors. The term “cancerous” denotes a malignant tumor that has the potential to spread and cause damage. Conversely, “non-cancerous” denotes a benign tumor that is incapable of disseminating and is not detrimental. The segmentation technique was employed to recover segments of the tumor images. Additionally, the authors implemented a median filter to completely eradicate any extraneous noise that was present in the background. Their model achieves an accuracy of 92.31% by employing a Classification and Regression Tree (CART) and a Support Vector Machine (SVM).

Within a comparable framework, Williams and Li (2018) presented a classification technique that employs wavelet pooling. The researchers found that wavelet pooling produced better results in comparison to other pooling methods. They obtained positive results, but, the amount of time required was significant. The effectiveness of a pooling strategy cannot be determined due to its reliance on multiple factors, such as the dataset and the number of levels used in different models.

The researchers in Zacharaki et al. (2009) proposed a technique that use a Support Vector Machine (SVM) to classify gliomas into distinct categories. Their multi-classification accuracy rate was 85%, while their binary classification accuracy rate was 88%. Furthermore, the authors in Machhale et al. (2015) presented a model that employs Support Vector Machines (SVM) to classify brain cancers. Additionally, they performed a comparison between two Convolutional Neural Network (CNN) models to determine the most efficient one in terms of attaining ideal results.

Babu et al. (2023) employed MRI images to develop a method for classification and segmenting brain tumors. The technique comprises four procedures: image denoising, tumor segmentation, feature extraction, and hybrid classification. After applying the thresholding technique to remove malignancies from brain MRI scans, they next utilized a wavelet-based approach to extract unique characteristics from the images. A Convolutional Neural Network (CNN) was utilized to carry out the conclusive hybrid categorization. The trial resulted in a segmentation accuracy of 95.23 percent for the technique, whereas the suggested optimized CNN attained a classification accuracy of 99 percent.

An advanced version of the Support Vector Machine (SVM) was proposed by Ansari (2023) as a novel approach. In order to identify and categorize brain cancers using MRI data, they suggested the implementation of the following four stages: preprocessing, image segmentation, feature extraction, and image categorization. The tumors were divided using a fuzzy clustering technique, and the fundamental characteristics were retrieved using GLCM. Improvements to the Support Vector Machine (SVM) were eventually integrated into the categorizing procedure. The technique used resulted in an accuracy rate of 88%.

### 2.2 Deep learning techniques

In order to gain a comprehensive understanding of deep learning algorithms in the specific context of brain tumor detection and diagnosis, it is crucial to consider the four fundamental deep learning tasks: single label image classification (İncir and Bozkurt, 2024b), multi-label/multi-class image classification, object detection in images, and dense prediction at the pixel level. The referenced study focuses on single label image classification. However, our objective is to achieve multi-label/multi-class image classification. This means that an input MRI scan can be assigned many tumor kinds or no tumor at all. An expansion of this work would involve identifying things inside an image, specifically cancers. Ultimately, dense prediction at the pixel level would entail accurately segmenting a tumor using MRI data.

Deep learning is a newly emerged field in machine learning that involves a classifier which receives an input x and converts it into a separate domain with the same dimensions. When it comes to annotated data classification tasks, the input domain refers to the collection of photos, while the output domain refers to the collection of class labels. This classifier can be seen as a combination of numerous elementary feature extraction and mapping modifications. Deep learning approaches have been minimally utilized in the domain of brain tumor detection using patient MRI data, but considerable progress has been made. This work employs a fusion between tow deep learning models, to classify input data into one of 44 distinct classes. Although this method achieves generally good classification outcomes, more sophisticated deep learning systems have the potential to effectively categorize complex and noisy data with high dimensionality into a reduced dimensional feature space, which could potentially lead to improved classification results.

In this context, Abd El Kader et al. (2021) introduced a convolutional neural network (CNN) method for classifying MR brain images. Their approach involved utilizing differential deep CNNs. In conventional Convolutional Neural Networks (CNNs), standard feature maps are generated through either random initialization or transfer learning. Nevertheless, this study generated distinct feature maps by utilizing user-defined hyperactive values and a differential operator proposed by Lei et al. (2018). The generated differential convolution maps are utilized to examine the directional patterns of voxels and their surrounding areas by computing the disparity in pixel activations. The study presented multiple data augmentation approaches to enhance the classification model's generalization performance. The process of data augmentation resulted in an expansion of the dataset, increasing its size to 25,000. The results indicate that the inclusion of differential feature maps enhanced the model's performance. The results additionally demonstrate that the suggested methodology may accurately classify a significant number of MR images. The approach attained a classification accuracy of 99.25%, sensitivity of 95.89%, and specificity of 93.75%.

A recent study conducted by Tanvir Rouf Shawon et al. (2023) introduced a cost-sensitive deep neural network (CS-DNN) designed to detect brain cancers from MRI (Magnetic Resonance Imaging) images. The suggested model employed a DenseNet architecture, a type of convolutional neural network, to automatically extract intricate and profound characteristics. Additionally, cost-sensitive learning was incorporated into the DenseNet to address the issue of class imbalance in the radiology dataset. The researchers employed both binary and multi-class cost-sensitive learning techniques to identify brain tumors from the MRI imaging dataset. CS-DNN outperformed seven alternative models in terms of sensitivity, specificity, precision, and accuracy for Tumor 3MRI images, making it the top performer.

Ge et al. (2020) addressed the issue of limited datasets by utilizing augmented brain MR images. A paired generative adversarial network (GAN) was employed to produce synthetic MR images for four different MRI techniques. The work involved extracting 2D MRI slices from three different perspectives of 3D volume images: coronal, axial, and sagittal. The 2D MRI slices that were obtained were separated into subgroups for training, validation, and testing purposes. Moreover, a paired Generative Adversarial Network (GAN) model was employed to produce artificial Magnetic Resonance Imaging (MRI) for the subset utilized in training. The pairwise GAN employed a dual input system with two separate streams. The purpose of this system is to address two specific situations: (a) generating artificial images of non-existent patients in order to expand the training dataset, and (b) generating artificial images for patients who are lacking certain MRI modalities. The study employed the U-Net architecture. The ultimate result of the architecture is the classification of glioma for each individual slice in a magnetic resonance (MR) image. Therefore, for every patient, the subtype of each MRI slice was taken into account, and the ultimate diagnostic or subtype categorization for the patient will be determined by a majority consensus. The experiments evaluated many case studies, and the case study that yielded the most favorable outcome attained an average classification accuracy, sensitivity, and specificity of 88.82%, 81.81%, and 92.17%, respectively.

### 2.3 Ensemble learning approach

Ensemble learning is employed to enhance the classification performance. Ensemble learning approaches leverage the power of several learning algorithms to achieve superior predicted performance compared to individual algorithms. Currently, there is a scarcity of research studies on brain tumor classification utilizing ensemble learning. Bansal and Jindal (2022) employed decision tree, random forest, and k-NN algorithms to classify brain tumors in MRI images. The classification of four types of cancers was performed using intensity, texture, and wavelet data. The highest level of accuracy achieved was 83.33% by utilizing the random forest algorithm.

Sekhar et al. (2021) introduced a tumor classification model that utilizes a modified GoogleNet pre-trained CNN model together with two machine learning algorithms: Support Vector Machine (SVM) and k-Nearest Neighbors (k-NN). The project involved modifying and fine-tuning the last three fully connected layers of the GoogleNet network using brain tumor photos. The 1,024 feature vector obtained from the final average pooling layer was recovered after fine-tuning and utilized for training SVM and k-NN classifiers. The method was assessed using the CE-MRI dataset, which consists of 3,064 T1w post GBCA brain MR images obtained from 233 patients. The experimental findings indicate that GoogleNet achieved a precision of 96.02% and a recall of 97.00% for glioblastoma while employing the softmax activation function. The utilization of the SVM classifier resulted in a performance enhancement of more than 2.5% for the model.

Jena et al. (2022) proposed a technique to classify and segment brain tumors by leveraging textural data and employing diverse machine learning algorithms. The technique comprises two separate stages: tumor categorization and tumor segmentation. During the tumor classification step, the MRI scans are subjected to pre-processing, and texture features are gathered from the images using several texture extraction methods. The study examined many features that are based on texture. Data was gathered from a combined set of 100 images of tumors and 100 images without tumors to extract features. The extracted characteristics were combined to form a feature vector matrix measuring 200 × 471. Subsequently, the feature vector matrix was employed to train five machine learning algorithms: Support Vector Machines (SVM), k-Nearest Neighbors (k-NN), binary decision trees, Random Forest (RF), and ensemble techniques. The ensemble methods consist of seven distinct algorithms: Adaboost, Gentleboost, Logitboost, LPboost, Robustboost, RUSboost, and Totalboost. After the training was finished, the study used the photos that had tumors to construct a hybrid technique for segmenting tumors. The hybrid technique involves combining the k-NN and fuzzy C-means clustering techniques. The hybrid approach was used to divide the tumor regions in the images. The dataset used for model evaluation consists of the BraTS2017 and BraTS2019 datasets, along with the Cancer Imaging Archive (TCIA). The experimental results indicate that the ensemble techniques achieved the maximum outcome.

Kang et al. (2021) introduced a technique for categorizing brain tumors by employing a combination of deep characteristics. The methodology comprises three distinct stages. During the initial phase, input photos undergo pre-processing, and additional images are created by data augmentation techniques. The preprocessed photos are subsequently utilized as input for 13 pre-trained convolutional neural network (CNN) models. Pre-trained convolutional neural network (CNN) models are employed to extract features from the images. Specifically, the characteristics are derived from the fully linked layers of the pre-trained models. The collected features are utilized for training nine machine learning classifiers, specifically: Gaussian Nave Bayes, Extreme Learning Machine (ELM), Adaptive Boosting (AdaBoost), k-NN, RF, SVM, and neural networks with a fully connected layer. Furthermore, the three most successful pre-trained models are identified, and the retrieved features from these models are merged into a single sequence. Ultimately, the amalgamated characteristics are employed to train the nine machine learning classifiers. The method was assessed using three brain MRI datasets obtained from Kaggle websites. The findings indicated that DenseNet-169, Inception-v3, and ResNeXt-50 offered the most favorable characteristics.

Deepak and Ameer (2021) introduced an automated technique for classifying brain tumors using Support Vector Machines (SVM) and Convolutional Neural Networks (CNN). A Convolutional Neural Network (CNN) was employed in the research to extract picture attributes from Magnetic Resonance Imaging (MRI) scans. The Convolutional Neural Network (CNN) comprises of five convolutional layers and two fully linked layers. The feature maps obtained from the fifth convolution layer and the first fully connected layer are isolated and utilized independently to train a Support Vector Machine (SVM) for the purpose of multiclass classification. The fifth convolution layer has 3,136 feature vectors, while the first fully linked layer has 10 feature vectors. When trained on the 10 feature vectors retrieved from the fully connected layer, the suggested approach attained an accuracy of 95.82%. The accuracy decreased to 93.83% when the model was trained using the 3,136-feature set retrieved from the fifth convolution layer. These findings indicate that Support Vector Machine (SVM) models trained on smaller feature sets have the capacity to yield superior outcomes compared to models trained on larger feature sets.

Multiple authors (Pereira et al., 2016) have investigated the use of tiny kernels to construct deeper networks that mitigate overfitting. The trials have shown that small kernels produce effective brain segmentation results. Furthermore, specific research has established effective and adaptable systems for the detection of brain cancers. Sharif et al. (2021) devised a versatile framework that can do multiple functions, including enhancing tumor visibility, extracting and selecting characteristics, localizing tumors, and segmenting tumors. The study included many advanced techniques such as the homomorphic wavelet filter, inception-v3 model, non-dominated sorted genetic algorithm (NGSA), YOLOv2, and ML algorithms to achieve specific objectives. These objectives included enhancing tumor visibility, extracting relevant features, selecting important features, localizing tumors, and segmenting tumors. The study's studies illustrate that the adaptable framework produced positive results. This method will be incredibly beneficial for medical practitioners as it allows them to effectively handle several responsibilities.

Binary classification is a significant obstacle for current classification algorithms. The majority of the current methodologies were devised to classify brain tumors into two distinct groups: benign and malignant. The study conducted by Sajjad et al. (2019) is among the limited number of research works that have devised a technique for classifying multiple classes simultaneously. Advanced multi-grade classification techniques have the potential to enhance the decision-making and diagnosing abilities of radiologists and other medical practitioners.

## 3 Proposed model

Convolutional neural networks (CNNs) have consistently achieved the highest level of performance in computer vision tasks, specifically in the areas of brain tumor segmentation and classification, in recent years. Nevertheless, Convolutional Neural Networks (CNNs) are limited in their ability to effectively collect extensive information or interconnections because of their tiny kernel size (Hatamizadeh et al., 2021). Long-range dependencies refer to situations when the desired outcome is influenced by visual sequences that were displayed at significantly earlier or later dates. Medical images often display a series of visual representations due to the resemblance of human organs (Dai et al., 2021). The elimination of these sequences will have a substantial impact on the performance of a CNN model. The reason for this is that the interconnections among medical picture sequences, such as modality, slice, and patch, provide substantial information (Dai et al., 2021). Sequences that have long-range dependencies can be effectively managed using techniques that are capable of processing sequence relations. The self-attention mechanism employed in ViTs (Dosovitskiy et al., 2020) possesses the ability to effectively capture long-range dependencies, a crucial factor in achieving accurate brain tumor segmentation. ViT-based models are able to learn local and global feature representations by modeling pairwise interactions between token embeddings, as described in Raghu et al. (2021)'s work on vision. ViT has exhibited encouraging performance on diverse benchmark datasets (Hatamizadeh et al., 2021; Wenxuan et al., 2021).

Although CNNs have achieved notable success, they do have certain limitations. Initially, Convolutional Neural Networks necessitate extensive datasets for the purpose of training. Furthermore, Convolutional Neural Networks (CNNs) generally lack resilience when it comes to affine rotations and transformations, as stated by Rodriguez et al. (2019) in their study on rotation. Moreover, the routing strategy utilized by CNN's pooling layers differs from the routing mechanism utilized by the human visual system. The CNN pooling layer distributes all the information acquired from the image to every neuron in the following layer, disregarding crucial details or little objects in the image (Aziz et al., 2021).

**Figure 1:**
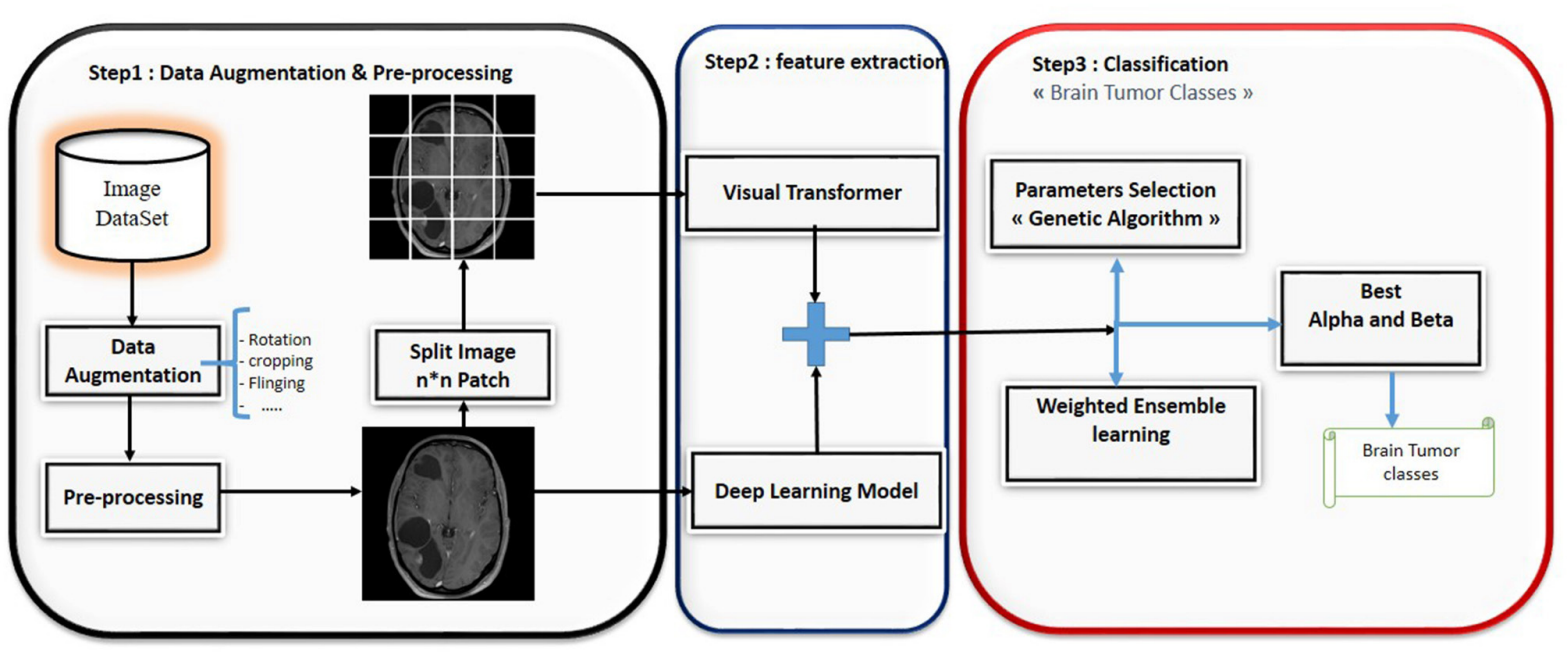
Hybrid deep learning approach for brain tumor classification.

This paper presents a novel ensemble learning method called BT-ViTEff, presented in Figure 1, which is designed for classifying brain tumors in medical MRI images. The approach aims to overcome challenging limitations such as complex and dynamic backgrounds that are caused by the presence of changing backgrounds in the input medical images. ViTEff integrates the advanced Convolutional Neural Network EfficientNet v2 (EfficientNetV2M) with the second version of the vision transformer. EfficientNet v2 is a novel convolutional neural network (CNN) architecture. The proposal aims to overcome the training constraint of EfficientNet models by demonstrating improved parameter efficiency and faster learning speed in comparison to comparable models. The system utilizes an enhanced progressive learning approach that dynamically modifies regularization approaches, including data augmentation and dropout algorithms, based on the input image size. In order to utilize EfficientNet v2 and ViT v2 models for brain tumor recognition, the classification layers (final layers) that were initially designed for distinct classification tasks are eliminated. As shown in Figure 1, the initial steps of preprocessing involve scaling the incoming RMI photos to dimensions of 224 × 224 pixels. Subsequently, a variety of data augmentation methods, such as rotation, shearing, shifting, and zooming, are employed to increase the diversity of the learning data. This enhances the capability of our model to accurately classify brain tumors into 44 distinct categories and prevents overfitting. Subsequently, the MRI images are inputted into the EfficientNet v2 and ViT v2 models to extract intricate features, thereby generating a comprehensive representation of different brain tumor classifications. The fusion model applies techniques such as Weighted Average Ensembling and Simple Average Ensembling after concatenating the feature maps generated by the EfficientNet v2 and ViT v2 models. This ensemble learning model will compute a probability classification for each image. Weighted ensembling is a form of model averaging ensembling, which falls under the domain of ensemble methods that aim to enhance prediction accuracy by aggregating the predictions of numerous models. Weighted ensembling involves assigning a specific weight or factor to each model's prediction, which represents the model's relative importance or performance. The weights allocated to the prediction of each model can be calculated using several strategies, including cross-validation, grid search, or meta-learning. Ultimately, we employ the genetic algorithm to determine the most ideal and superior weight for each deep learning model utilized.

### 3.1 EfficientNet model

EfficientNet utilizes a compound coefficient to scale the size of a CNN (İncir and Bozkurt, 2024c). The EfficientNet scaling approach employs a standardized set of scaling coefficients to evenly change these values, hence enhancing the standard procedure. By increasing the network depth by a factor of α, β, and γ, we are able to utilize a total of 2N times the quantity of processors that are currently accessible. By conducting a rapid grid search on the initial, simplified model, we obtained these fixed coefficients. If the input image is larger, it is logical to conclude that a network requires extra layers and channels to expand the receptive field and capture more detailed patterns. The EfficientNet-B0 network was built by incorporating MobileNetV2's inverted bottleneck residual blocks and incorporating additional squeeze-and-excitation blocks.

### 3.2 Vision transformer model

The Transformer architecture, presented in Vaswani et al. (2017), is currently at the forefront of new research in natural language processing (NLP). Dosovitskiy et al. (2021) was inspired by the success of self-attention-based deep neural networks in natural language processing to design the Vision Transformer (ViT) architecture for picture categorization in NLP. Training these models often requires decomposing the input image into its individual components and subsequently considering each embedded component as if it were a word in a natural language processing (NLP) system. These models utilize self-observation modules to establish the connection between the concealed patches. Due to their exceptional efficacy, numerous scientists have investigated ViT models for diverse visual tasks (Yu et al., 2021). Carion et al. (2020) introduced a novel architecture for object recognition systems. This architecture utilizes an aset-based global loss and a transformer-encoder-decoder technique. They obtained equivalent results to the popular R-CNN approach on the challenging COCO dataset.

**Figure 2:**
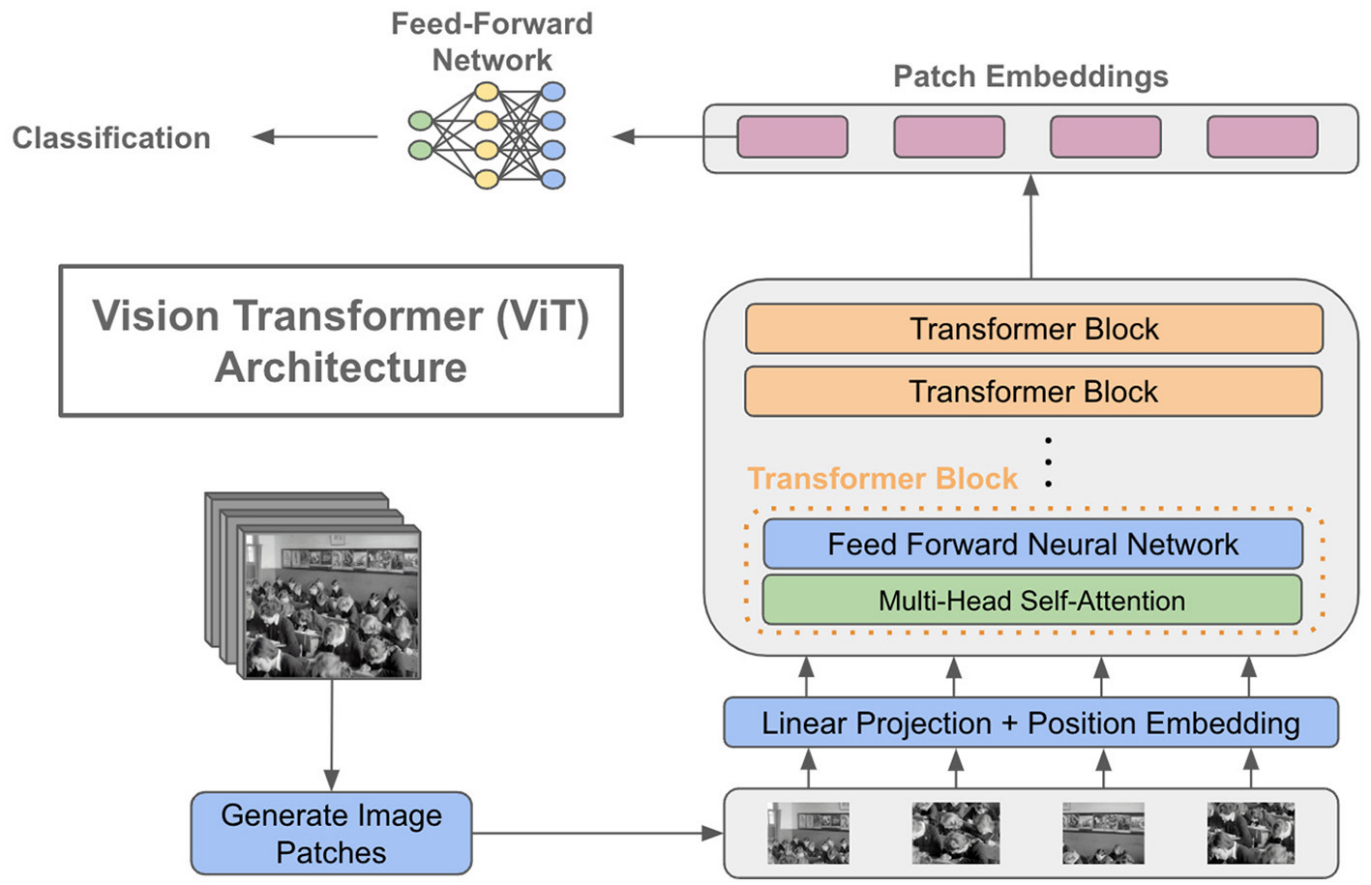
A basic depiction of a vision transformer architecture. ^a^https://cameronrwolfe.substack.com/p/vision-transformers.

Steiner et al. (2021) initially presented his established architecture, which closely resembled the original ViT design by Dovosviky, but with a linear classifier instead of the MLP header. To summarize, the initial stage of training a ViT model involves dividing the input image into smaller segments. The transformer encoder takes a sequence of 1D patch embeddings as input. It employs self-attention mechanisms to compute a weighted sum of the outputs from each hidden layer, considering their interdependencies. This is accomplished by feeding the sequence into an encoder. The transformers utilize this mechanism to decode the global dependencies of the input photos. Refer to Figure 2 for a simple vision 348 transformer architecture.

### 3.3 Ensemble learning for brain tumor classification

At this point, we were concentrating on the last step to classify the input image. Ensemble learning models strive to combine the outcomes of several algorithms to improve overall performance and facilitate interpretation. This section highlights the hybridization strategy commonly employed to enhance predictive accuracy and resilience. Numerous ensemble learning techniques are documented in the literature, and we will use two approach:

Ensemble averaging is a technique used to enhance the accuracy and stability of a predictive model by merging the predictions of numerous base models. In this methodology, every foundational model is trained using the identical dataset, but with distinct hyperparameters, algorithms, or subsets of features. This is done to capture various facets of the data and mitigate the likelihood of overfitting. After training the base models, their forecasts are consolidated into a single prediction by an averaging strategy, such as simple averaging or weighted averaging. The arithmetic mean is determined by summing the forecasts of each base model and thereafter dividing by the total number of models. Simple average ensembling is a direct and efficient method for merging the predictions of many models, particularly when the separate models exhibit comparable performance and reliability.

Simple average ensembling is a versatile technique that may be used in various machine learning applications and algorithms, including as regression, classification, and clustering, among others. In addition, it is possible to integrate this strategy with other ensemble methods, such as weighted averaging or bagging, in order to enhance the model's performance and accuracy. Weighted ensembling involves assigning a specific weight or factor to each model's prediction, which indicates the model's relative importance or performance. The weights allocated to the prediction of each model can be established by many strategies, including cross-validation, grid search, or meta-learning.

Weighted ensembling is a versatile and potent strategy that can merge the advantages of several models and alleviate their limitations, resulting in enhanced prediction accuracy and resilience. In our model, we use an optimal algorithm to select the optimal weight for each model.

### 3.4 Optimal weight selection

In the domain of medical image classification, achieving high accuracy is paramount and ensemble learning, which combines the predictions from multiple models, often yields superior performance compared to individual models; however, determining the optimal weights for each model in the ensemble is a challenging task and to address this, we propose the use of a Genetic Algorithm (GA) for weight selection, aiming to maximize the accuracy of our brain tumor classification system; a Genetic Algorithm is an optimization technique inspired by the principles of natural selection and genetics, operating through a process of selection, crossover, and mutation to evolve solutions toward optimality, with the main components including a population of potential solutions (chromosomes) to the optimization problem, each chromosome representing a pair of weights alpha and beta for the deep learning models in the ensemble, and a fitness function evaluating how well each chromosome solves the problem, which in our case is the accuracy of the ensemble model; the selection process chooses the best chromosomes for reproduction based on their fitness scores, with crossover combining pairs of selected chromosomes to produce offspring, and mutation introducing random variations to maintain genetic diversity, all iterating through generations until convergence criteria are met, such as a maximum number of generations or a satisfactory fitness score; the implementation of the GA involves generating an initial population with random values for alpha and beta, evaluating the fitness function for each chromosome based on the accuracy of the ensemble model, selecting the top-performing chromosomes, performing crossover and mutation to create a new generation, and repeating the process until the optimal weights are found, thus leveraging the complementary strengths of the Vision Transformer and other deep learning models, with results demonstrating that the GA-enhanced weighted average ensemble outperforms individual models and simple averaging methods, achieving superior accuracy, precision, recall, and F1-score, ultimately contributing to more reliable and accurate diagnostic tools in healthcare, and highlighting the potential of evolutionary algorithms in fine-tuning ensemble learning models for complex medical image classification tasks.

**Figure 3:**
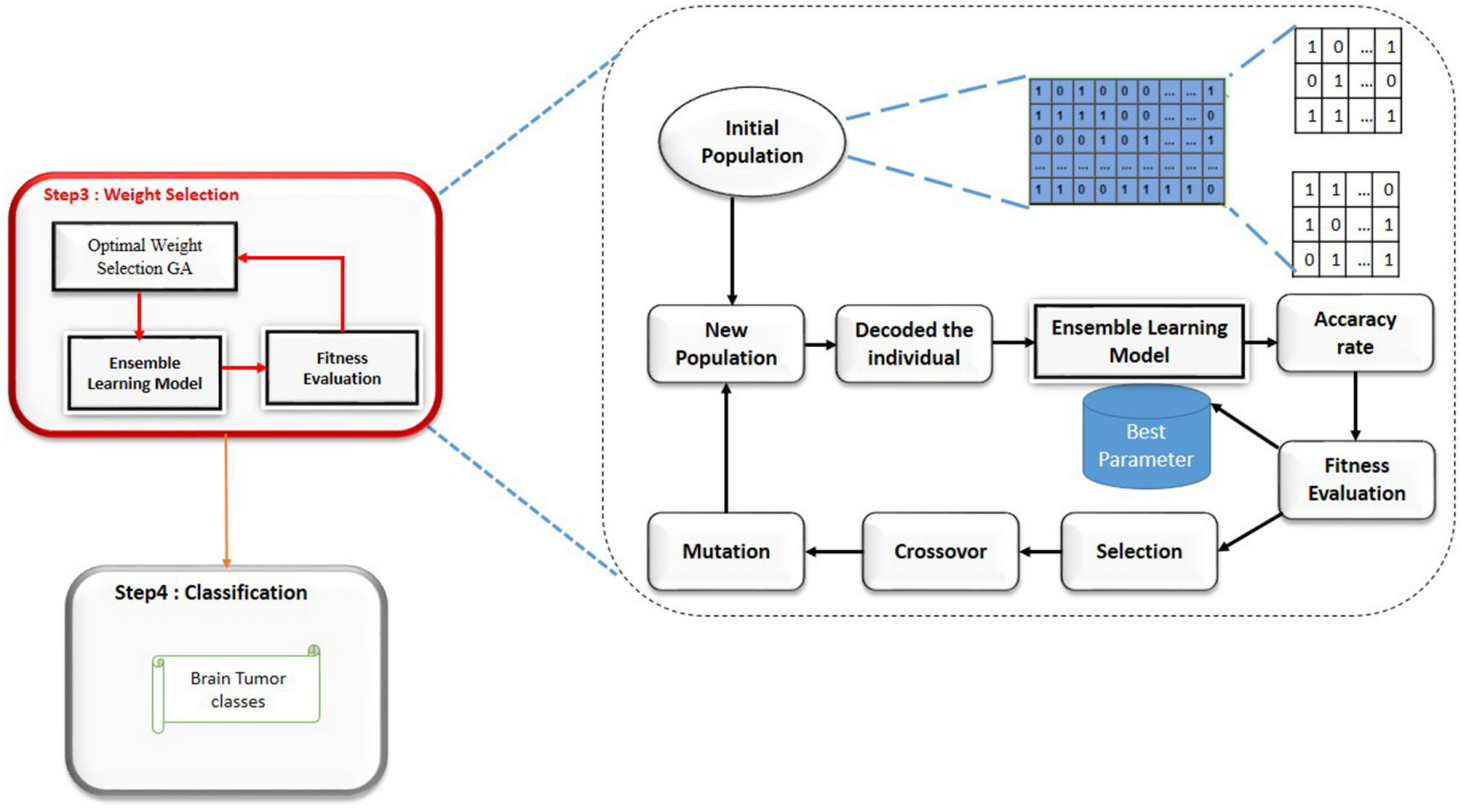
Genetic algorithm steps (Gasmi et al., 2024).

To adapt the Genetic Algorithm for selecting the optimal weights (alpha and beta) in our ensemble learning model, we follow these steps, Figure 3: 1) Chromosome Representation: Each chromosome represents a pair of weights α and β for the models in the ensemble. The weights must satisfy the condition : α and β = 1 2) Initial Population: Generate an initial population of chromosomes with random values for α and β.

3) Fitness Function: The fitness function evaluates the accuracy of the ensemble model for each chromosome. Given a chromosome with weights α and β, the fitness function is defined as: Fitness(α, β)=Accuracy;

4) Selection: Select the top-performing chromosomes based on their fitness scores. Techniques such as roulette wheel selection or tournament selection can be used.

5) Crossover: Combine pairs of selected chromosomes to produce offspring. For example, a simple crossover method could be:

Offspring1 = (α_1_, β_2_) and Offspring2 = (α_2_, β_1_)

where (α_1_, β_2_) and (α_2_, β_1_) are parent chromosomes6) Mutation: Introduce random changes to the offspring to maintain genetic diversity. For instance, a small random value could be added or subtracted from (α, β), ensuring that the sum remains 1.

7) New Generation: Replace the old population with the new generation of chromosomes.8) Termination: Repeat the selection, crossover, and mutation steps until convergence criteria are met, such as a maximum number of generations or a satisfactory fitness score.

### 3.5 Results and discussions

This section provides the findings of the experiments conducted on the model described in this study. In addition, we evaluate the outcomes achieved through various classification techniques using deep learning models. The Python implementation of the suggested model utilized a Rtx 2,060 graphics card and 16 GB of RAM.

In order to implement our suggested model, we conducted multiple sets of tests utilizing deep learning models. The structure of our model encompasses three distinct scenarios:

1. Scenario 1: Brain Tumor classification based on the deep learning models.
2. Scenario 2: Brain Tumor classification based on the hybrid deep learning models.
3. Scenario 3: Brain tumor classification based in the weight selection method.

### 3.6 Data set description and evaluation metrics

**Figure 4:**
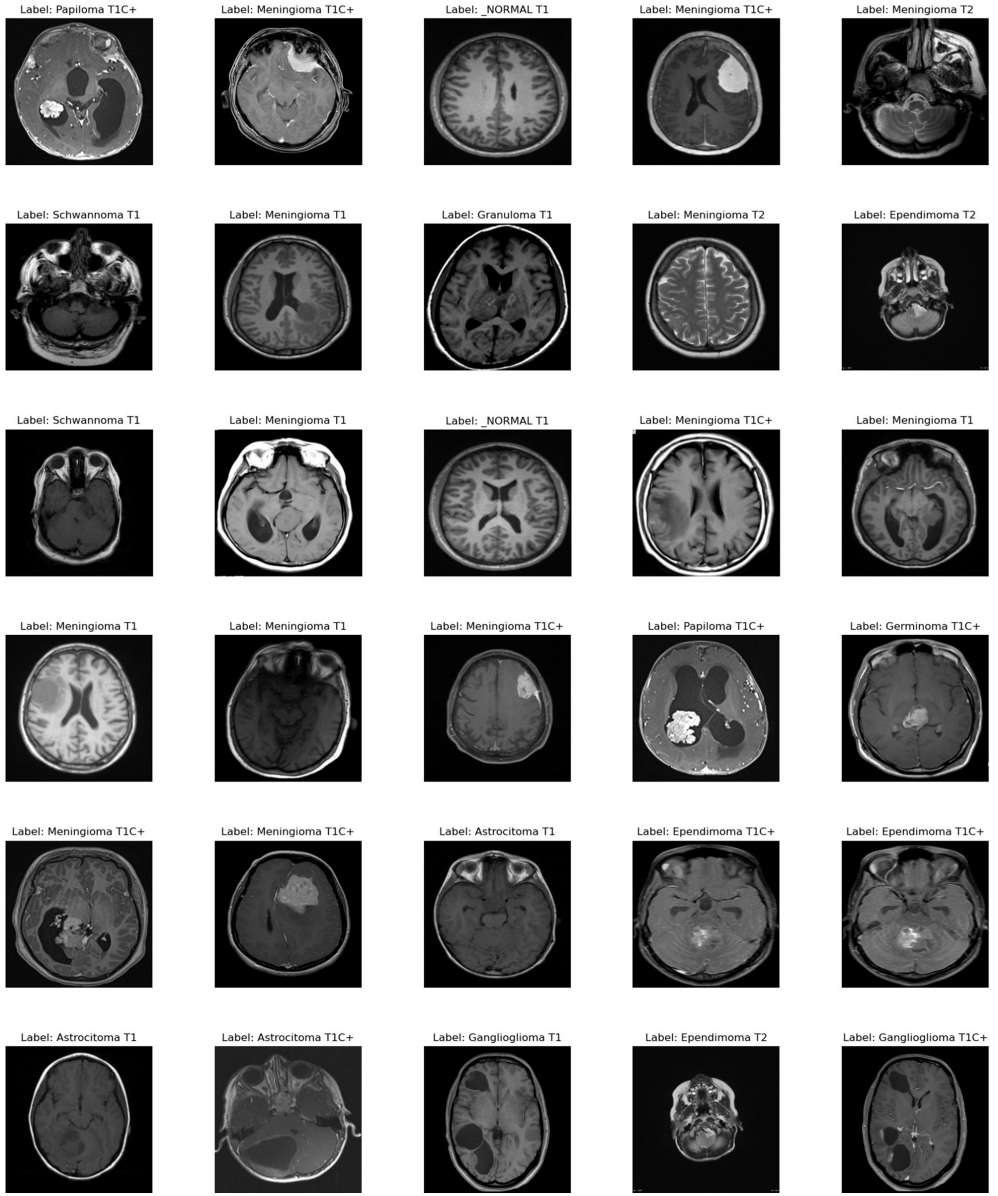
Examples of images taken from the dataset.

To assess our improved deep learning model, we used a dataset with 44 classes,^1^ which is available online. Figure 4 provides an example of each classes in the data set.

We employed typical measures, such as accuracy, precision, recall, and F-measure, to test our classification approach.


(1) $$\begin{array}{l} Accuracy=\left(TP+TN\right)/\left(TP+TN+FP+fN\right) \end{array}$$

(2) $$\begin{array}{l} Precision=TP/\left(TP+FP\right) \end{array}$$

(3) $$\begin{array}{l} Recall=TP/\left(TP+FN\right) \end{array}$$

(4) $$\begin{array}{l} F1-score=TP/\left(TP+0.5*\left(FN+FP\right)\right) \end{array}$$

Where TP stands for true positive, FP stands for false positive, P stands for precision, R stands for recall, TPR stands for true positive rate, and FPR stands for false positive rate.

### 3.7 Brain tumor classification based on deep learning models

The main objective of this study was to utilize hybrid deep learning models for the categorization of brain tumors. However, in this section, we aimed to classify MRI images into 44 distinct classes of brain tumors by leveraging various state-of-the-art deep learning models. The models evaluated in this study include Inception-ResNet-v2, EfficientNet V2, MobileNet V2, and the Vision Transformer (ViT). These models were chosen because of their strong track record in image classification, particularly in medical imaging. They offer a good balance between high accuracy and computational efficiency, making them ideal for complex tasks like brain tumor classification. The decision to use these models was based on results from similar studies in the same field, where they consistently performed well. Each model brings unique strengths: Inception-ResNet-v2 is excellent at capturing detailed features in images, EfficientNet V2 is highly efficient and scalable, MobileNet V2 is lightweight and suited for mobile applications, and ViT uses transformer-based attention mechanisms to extract deep features. Together, these models form a strong foundation for evaluating and improving tumor classification using medical image datasets. Our results, as detailed in Table 1, demonstrate significant variation in performance metrics such as accuracy, precision, recall, and F1-score across the different models.

**Table 1:** Performance metrics for different deep learning models.

	**Accuracy**	**Precision**	**Recall**	**f1_score**
**Inception-resnet-v2**	0.708185	0.733747	0.708185	0.710366
**Efficientnet-v2**	**0.939502**	**0.950218**	**0.939502**	**0.940100**
**MobileNet-V2**	0.740214	0.773633	0.740214	0.738958
**Vision transformer**	0.879004	0.896360	0.879004	0.881115

From the results, EfficientNet V2 emerges as the top-performing model with an accuracy of 93.95%, precision of 95.02%, recall of 93.95%, and F1-score of 94.01%. This superior performance can be attributed to EfficientNet V2's optimized architecture, which balances depth, width, and resolution for better accuracy and efficiency.

The Vision Transformer (ViT B16) also performed remarkably well, achieving an accuracy of 87.90%, precision of 89.64%, recall of 87.90%, and F1-score of 88.11%. This indicates that the ViT model, with its ability to capture global dependencies in the image data, is well-suited for complex image classification tasks such as brain tumor classification.

In contrast, the Inception-ResNet-v2 and MobileNet V2 models demonstrated lower performance metrics. The Inception-ResNet-v2 model achieved an accuracy of 70.82%, precision of 73.37%, recall of 70.82%, and F1-score of 71.04%. MobileNet V2, known for its lightweight architecture, attained an accuracy of 74.02%, precision of 77.36%, recall of 74.02%, and F1-score of 73.90%. While these models are efficient, their performance is not as robust as EfficientNet V2 and ViT for this specific task.

The discrepancy in performance can be attributed to several factors. EfficientNet V2's scaling strategy allows it to adaptively scale network dimensions, leading to better feature extraction and classification accuracy. ViT's attention mechanisms enable it to handle the spatial hierarchies and complex patterns in MRI images more effectively. On the other hand, while Inception-ResNet-v2 combines the strengths of Inception and ResNet architectures, it might not be as finely tuned for this specific application. Similarly, MobileNet V2's design for mobile and edge devices might limit its capacity to capture intricate details necessary for distinguishing between 44 brain tumor classes.

In conclusion, our findings underscore the importance of model selection in medical image classification tasks. EfficientNet V2 and Vision Transformer (ViT) stand out as particularly effective for classifying brain tumors from MRI images. Future work could focus on further optimizing these models and exploring ensemble methods to enhance classification performance even further. Additionally, incorporating domain-specific data augmentation and preprocessing techniques could potentially improve the accuracy and reliability of the classification results.

### 3.8 Brain tumor classification based on an hybrid deep learning model

In our study, we aimed to classify MRI images into 44 distinct classes of brain tumors using various state-of-the-art deep learning models. To further enhance classification performance, we employed ensemble learning techniques, combining the Vision Transformer (ViT) model with other models such as Inception-ResNet-V2, EfficientNet V2, and MobileNet V2. The ensemble methods included both average and weighted average techniques, with the latter varying the weights (alpha for the first model and beta for ViT) to optimize performance. Table 2 below summarizes the results.

**Table 2:** Performance metrics for hybrid models.

**Hybrid model**	**Ensemble learning technique**	**Accuracy**	**Precision**	**Recall**	**f1_score**
**ViT + InceptionResnet V2**	Average Ensemble	0.886121	0.903569	0.886121	0.888955
	Weighted average: (α = 0.2, β = 0.8)	0.900356	0.908926	0.900356	0.900590
	Weighted average: (α = 0.8, β = 0.2	0.779359	0.802382	0.779359	0.781597
**ViT + MobileNet V2**	Weighted average: (α = 0.2, β = 0.8)	0.882562	0.892685	0.882562	0.882396
	Weighted Average: (α = 0.8, β = 0.2)	0.786477	0.805635	0.786477	0.781143
**ViT + EfficientNet V2**	Average Ensemble	***0.953737***	***0.961463***	***0.953737***	***0.954080***
	Weighted Average: (α = 0.8, β = 0.2)	***0.950178***	***0.961736***	***0.950178***	***0.950381***
	Weighted Average: (α = 0.2, β = 0.8)	0.939502	0.952808	0.939502	0.941447

From the results, the combination of ViT and EfficientNet-V2 using average ensemble learning achieved the highest performance with an accuracy of 95.37%, precision of 96.15%, recall of 95.37%, and F1-score of 95.41%. This demonstrates the robustness of ensemble methods in leveraging the strengths of different models to enhance classification performance.

The weighted average ensemble also showed promising results, particularly with a weight distribution of α = 0.2 and β = 0.8 for the ViT and EfficientNet-V2 combination, achieving an accuracy of 93.95%. This suggests that placing more emphasis on the ViT model, which effectively captures global dependencies, can significantly boost performance.

Interestingly, the ViT and Inception-ResNet-V2 combination also performed well, especially with the weighted average ensemble method (α = 0.2, β = 0.8), achieving an accuracy of 90.04%. However, the performance dropped when the weight distribution favored the Inception-ResNet-V2 model (α = 0.8, β = 0.2), indicating the importance of optimal weight selection in ensemble methods.

Similarly, the combination of ViT and MobileNet-V2 showed improved performance with the weighted average method (α = 0.2, β = 0.8), achieving an accuracy of 88.25%, while favoring MobileNet-V2 (α = 0.8, β = 0.2) resulted in lower performance.

In summary, our findings highlight the efficacy of ensemble learning in brain tumor classification. By integrating different models and optimizing weight distributions, we can significantly improve classification accuracy and other performance metrics. The success of the ViT and EfficientNet-V2 combination, in particular, underscores the potential of using advanced deep learning models and ensemble techniques for complex medical image classification tasks. Future work could explore further optimizations and the incorporation of additional models to continue enhancing classification performance.

### 3.9 Evaluation of the significance improvement of ensemble learning by weighted selection method for brain tumor classification

In this section, we evaluate the significance of our optimization method in improving the performance of ensemble learning for brain tumor classification. By applying a genetic algorithm to determine the optimal weights for combining deep learning models, we aimed to enhance classification accuracy, precision, recall, and F1-score. The results in Table 3 demonstrate the effectiveness of our approach, highlighting the potential of weighted ensemble learning in advancing medical image classification tasks.

**Table 3:** Performance metrics of optimized weighted average models for brain tumor classification using genetic algorithm-selected alpha (α) and beta (β) values.

	**Accuracy**	**Precision**	**Recall**	**f1_score**	**top_3_accuracy**
**Weighted average (α** **= 0.8**, **β** **= 0.2)**	0.950178	0.959987	0.950178	0.951458	0.982206
**Weighted average (α** **= 0.44**, **β** **= 0.56)**	0.9609	0.9691	0.9609	0.9616	0.9822
**Weighted average (α** **= 0.17**, **β** **= 0.83)**	0.9395	0.9530	0.9395	0.9415	0.9822
**Weighted average (α** **= 0.73**, **β** **= 0.27)**	0.9609	0.9673	0.9609	0.9610	0.9822

The application of a genetic algorithm to optimize the weights (alpha and beta) for combining deep learning models demonstrated significant improvements in performance metrics for brain tumor classification. The results shown in the Table 3 illustrate the efficacy of the weighted average ensemble learning approach across multiple generations, where each generation represents a different combination of alpha (α) and beta (β) values. The optimized weighted average model with α = 0.44 and β = 0.56 achieved the highest accuracy score of 0.9609, coupled with a precision score of 0.9691, indicating that the genetic algorithm successfully identified a near-optimal balance between the two models, enhancing the overall accuracy and precision of the classification. Across different generations, the results consistently show high values for accuracy, precision, recall, and F1-score, underlining the robustness of the genetic algorithm in optimizing the ensemble weights. For instance, models with α = 0.44, β = 0.56 and α = 0.73, β = 0.27 both achieved an accuracy of 0.9609, reflecting reliable performance improvements. The impact of varying the weights is evident, as seen in the model with α = 0.17, β = 0.83, which achieved a slightly lower accuracy of 0.9395, emphasizing the importance of fine-tuning the weights to leverage the strengths of each model optimally. The top-3 accuracy metric remains consistently high (0.9822) across different weight combinations, indicating the model's robustness and reliability in clinical scenarios. The enhanced performance metrics obtained through the genetic algorithm optimization have significant implications for clinical practice, ensuring more reliable diagnoses critical for treatment planning and patient outcomes, thus providing confidence in the model's predictions and aiding clinicians in making more informed decisions.

### 3.10 Comparative study

The Table 4 compares our work to several other studies on brain tumor classification, highlighting differences in the methods used, the datasets, the number of classes, and the resulting accuracy. A key point of comparison is the complexity of the datasets, particularly the number of classes, which has a significant impact on the difficulty of the classification task.

**Table 4:** Comparative study between proposed approaches for brain tumor classification.

**Study**	**Method**	**Dataset**	**Number of class**	**Accuracy**
Abiwinanda et al. (2019)	CNN	^1^	3	88.86
Sajjad et al. (2019)	VGG-16	–	3	94.58
Deepak and Ameer (2021)	CNN+SVM	–	3	95.82
Asif et al. (2023)	Xception	^2^	4	95.87
İncir and Bozkurt (2024a)	EfficientNetV2-M + Inception-V3	–	4	98.41
Sandhiya and Raja (2024)	ELM+PSO	–	4	97.97
Vankdothu et al. (2022)	LSTM+CNN	–	4	92
**Our work**	**ViT+ EfficientNetV2**	**1**	**44**	**96.09**

^1^https://figshare.com/articles/dataset/brain_tumor_dataset/1512427. ^2^https://www.kaggle.com/datasets/sartajbhuvaji/brain-tumor-classification-mri.

In earlier studies, such as those proposed by Avants et al. (2008); Hinton and Sejnowski (1986), the datasets consist of only three classes. These studies achieved high accuracies, ranging from 88.86% to 95.82%, using CNN-based models. However, with fewer classes, the task is relatively simpler, as the models have fewer categories to differentiate between, making high accuracy more achievable.

Authors in Tomasila and Emanuel (2020), are worked with a dataset of four classes, comprising 53 images per class, and reported accuracies between 92% and 98.41%. The highest accuracy in this group, 98.41%, was achieved by reviewer, who used a combination of EfficientNetV2-M and Inception-V3.

In contrast, our study takes on a much more complex task, utilizing a dataset with 44 classes, significantly increasing the difficulty of the classification problem. Despite this challenge, our model–combining Vision Transformers (ViT) with EfficientNetV2–achieved an accuracy of 96.09%. This is a remarkable result, considering the higher number of classes. Our model needed to differentiate between many more categories, each with potentially subtle differences, making the classification process more demanding.

While some studies report slightly higher accuracy, it's important to recognize that they were working with much simpler datasets. Our model's ability to maintain a high accuracy on a dataset with 44 classes demonstrates its robustness and ability to handle more complex tasks. This makes our approach particularly valuable for real-world applications where models need to classify a wide range of tumor types or conditions with high precision.

## 4 Conclusion

In this study, we have explored the efficacy of combining different deep learning models, including Inception-ResNet-V2, EfficientNet-V2, MobileNet V2, and Vision Transformers (ViT), for the classification of MRI images into 44 brain tumor classes. Our experiments demonstrated that ensemble learning techniques, particularly weighted average ensembles, significantly enhance classification performance, with the ViT + EfficientNet-V2 model achieving the highest accuracy. Furthermore, by adopting a Genetic Algorithm to optimize the weights in the ensemble models, we were able to achieve even greater improvements in accuracy, precision, recall, and F1-score. These results underscore the potential of hybrid deep learning approaches and optimization algorithms in advancing medical image classification. Future work will focus on further refining these models and exploring their applicability to other complex medical imaging tasks, ultimately contributing to more accurate and reliable diagnostic tools in healthcare.

## Data Availability

The datasets presented in this study can be found in online repositories. The names of the repository/repositories and accession number(s) can be found below: https://www.kaggle.com/datasets/fernando2rad/brain-tumor-mri-images-44c.

## Footnotes

^1^ https://www.kaggle.com/datasets/fernando2rad/brain-tumor-mri-images-44c
